# Sulphate-reducing bacterial community structure from produced water of the Periquito and Galo de Campina onshore oilfields in Brazil

**DOI:** 10.1038/s41598-021-99196-x

**Published:** 2021-10-13

**Authors:** Samyra Raquel Gonçalves Tiburcio, Andrew Macrae, Raquel Silva Peixoto, Caio Tavora Coelho da Costa Rachid, Felipe Raposo Passos Mansoldo, Daniela Sales Alviano, Celuta Sales Alviano, Davis Fernandes Ferreira, Fabrício de Queiroz Venâncio, Doneivan Fernandes Ferreira, Alane Beatriz Vermelho

**Affiliations:** 1 Post Graduate Program in Plant Biotechnology and Bioprocesses, Decania, Center for Health Sciences, Universidade Federal do Rio de Janeiro (UFRJ), Rio de Janeiro, Brazil; 2 Institute of Microbiology Paulo de Góes, Brasil, Universidade Federal do Rio de Janeiro (UFRJ), Rio de Janeiro, Brazil; 3 Red Sea Research Center (RSRC), Division of Biological and Environmental Science and Engineering (BESE), King Abdullah University of Science and Technology (KAUST), Thuwal, Saudi Arabia; 4 Department of Molecular and Structural Biochemistry, North Carolina State University, Raleigh, NC USA; 5 Capital Intelectual Instituto Interdisciplinar de P&D (C3i), Limeira, SP Brazil; 6 BIOINOVAR - Biocatalysis, Bioproducts and Bioenergy Lab, Universidade Federal do Rio de Janeiro (UFRJ), Rio de Janeiro, Brazil

**Keywords:** Biodiversity, Bioinformatics, PCR-based techniques, Biotechnology, Ecology, Microbiology, Biogeochemistry, Ecology, Environmental sciences, Ocean sciences, Biomarkers, Chemistry, Energy science and technology

## Abstract

Sulphate-reducing bacteria (SRB) cause fouling, souring, corrosion and produce H_2_S during oil and gas production. Produced water obtained from Periquito (PQO) and Galo de Campina (GC) onshore oilfields in Brazil was investigated for SRB. Produced water with Postgate B, Postgate C and Baars media was incubated anaerobically for 20 days. DNA was extracted, 16S rDNA PCR amplified and fragments were sequenced using Illumina TruSeq. 4.2 million sequence reads were analysed and deposited at NCBI SAR accession number SRP149784. No significant differences in microbial community composition could be attributed to the different media but significant differences in the SRB were observed between the two oil fields. The dominant bacterial orders detected from both oilfields were Desulfovibrionales, Pseudomonadales and Enterobacteriales. The genus *Pseudomonas* was found predominantly in the GC oilfield and *Pleomorphominas* and *Shewanella* were features of the PQO oilfield. 11% and 7.6% of the sequences at GC and PQO were not classified at the genus level but could be partially identified at the order level. Relative abundances changed for *Desulfovibrio* from 29.8% at PQO to 16.1% at GC. *Clostridium* varied from 2.8% at PQO and 2.4% at GC. These data provide the first description of SRB from onshore produced water in Brazil and reinforce the importance of Desulfovibrionales, Pseudomonadales, and Enterobacteriales in produced water globally. Identifying potentially harmful microbes is an important first step in developing microbial solutions that prevent their proliferation.

## Introduction

Petroleum is found in reservoir rocks typically between a gas cap at the top and water at the bottom^1^. Petroleum, and gas are recovered from low permeability formations through horizontal drilling and hydraulic fracturing technologies that release viable amounts of oil and gas^2–4^. Oil extracted from a reservoir rock reaches the surface as a mixture with some sediment, gas and water. Water naturally confined in a reservoir is called Formation Water; after it has been extracted with oil and gas, it is called Produced Water^5^. Due to technical considerations (equipment and reservoir) and environmental legislation, produced water requires special attention and specific management actions^6,7^. Oil from a reservoir is pumped through pipelines to a primary processing unit, where density is used to separate particles, water, oil and gas and the emulsified zone between the oil and water. At the beginning of the operation, the liquid phase is separated from the gas and water and then the emulsified zone is separated from the oil phase^8^. Before disposing of, or injecting the produced water back into a well, the operator must treat it to meet physical, chemical and biological quality control legislation that aims to protect the natural environment and to prevent fouling of the reservoir. If an operator decides to reinject the water into the reservoir, it is treated for bacteria, with biocide. Bacteria can create integrity problems for assets and safety problems for personnel^9^. Salts and solid/rock particles can create flow problems causing strangulation within the tubes and pipes. Solids will also create permeability problems within the reservoir rock^10^. Regulations covering the management and fate of produced water are increasingly stringent but not globally standardised. Treatment is required before disposing of produced water to remove any remaining oil and grease. In Brazil, produced water is legally regarded as an “effluent” by the National Environmental Council (CONAMA). CONAMA Resolution No. 357/2005 and its amendments (393/2007 and 397/2008) establish acceptable oil limits in produced water but do not mention microbial contamination. Qualitative and quantitative characterization of the bacteria in produced water has not been a focus of studies in Brazil. It is interesting to identify bacteria present in produced water as a proxy for communities in the reservoir and to detect harmful bacteria such as sulphate reducing bacteria (SRB). If we can identify specific SRB this knowledge is an important step in the choice and management of biocides for microbial control. It is accepted that microorganisms are introduced into the reservoir during the drilling process, and/or by the injection of produced water or naturally through venting with ocean water offshore and ground water onshore^11^. In these instances, the microbial communities are a combination of native microorganisms in the well, and microorganisms present at the surface portion of the well added by drilling or injection^12,13^. Oil production faces four microbially attenuated SRB problems and they include: (i) microbial induced corrosion (MIC) in and on pipelines, metal structures and equipment^14^; (ii) contamination of injection wells; (iii) impact of bioaccumulation (biofouling) on production and flow rates^15^; and (iv) biogenic acidification (souring), which is caused by sulphide production in the form of Hydrogen Sulphide (H_2_S)^16^. In addition, hydrogen sulphide is an acidic gas and thus represents a risk for on-site personal^17,18^. To control these problems, operators use biocides. Without microbiological monitoring and resistance studies to assess biocide efficacy and impact, these efforts only result in limited success^19^. In this context, research that seeks new technologies and new biocidal substances must be combined with microbiological studies. Numerous studies on biofilms show that most biocides only have limited penetration capabilities, as they kill mostly surface cells and are more effective against planktonic cells^20–22^. There is also evidence of active microbial responses to biocides leading to transcriptionally regulated biocide enhanced resistance in produced water settings. Vikram et al^19^ describe the occurrence of genetic, and multifactorial regulation mechanisms of glutaraldehyde resistance.

Accurate knowledge of the microorganisms present in an oil field and its produced water is critical for selecting and monitoring the action of antimicrobial substances. It is also critical in order to establish the required/optimum amount of biocide, its duration of use, and stage of application. It is necessary to stop biofilms at an early stage of establishment because treating them subsequently is much more challenging.

A wide variety of SRB and other microorganisms have been isolated or detected in samples from oil reservoirs and produced water, including aerobic bacteria, facultative anaerobes, microaerophilic bacteria, strict anaerobes, archaea, thermophilic organisms, mesophilic and hyper thermophilic organisms^12^. Some functional groups of microorganisms such as the SRB are frequently described as present. Other bacteria routinely found in reservoirs include members of genera *Clostridium*, *Pseudomonas* and *Bacillus* and other groups include the Acid Producing Bacteria, APB^23^, Oxidizing Iron Bacteria, OIB^24^, and Methanogenic Archaea (MA)^25–29^. Petrobras’s studies on bacterial communities and especially SRB in produced water are ongoing and to date bacterial strains from the following genera have been isolated from Brazilian oilfields *Curtobacterium*, *Brevundimonas*, *Brachymonas*, *Streptomyces*, *Bacillus*, *Pseudomonas* amongst others^30,31^. Bacteria belonging to the Firmicutes are commonly isolated and within them the Bacilli and Clostridia are most often reported. Members of the genera *Marinobacter*, *Colwellia* and *Pseudomonas* have been associated with corrosion and biofilms in oil pipelines*.* Strains from these genera are known to stimulate corrosion by producing large amounts of extracellular polysaccharides that protect SRB from biocides. Isolates from these genera have been shown to contribute to corrosion and to produce hydrogen sulphide impacting negatively on the market value of the crude oil and a threat to staff safety^30–34^.

In this study, we used Next Generation Sequencing (NGS) methods to study produced water that had been enriched for SRB from two Brazilian oilfields: Galo de Campina and Periquito, both located in Rio Grande do Norte. Very little was known about the microbiology of these wells. These studies are rare in Brazil despite the country's important role in the oil industry. Molecular biology and NGS methods were used to investigate samples and to establish a diversity benchmark as well as identifying potential biomarkers for the oilfields studied.

## Materials and methods

### Sample collection

**Figure 1:**
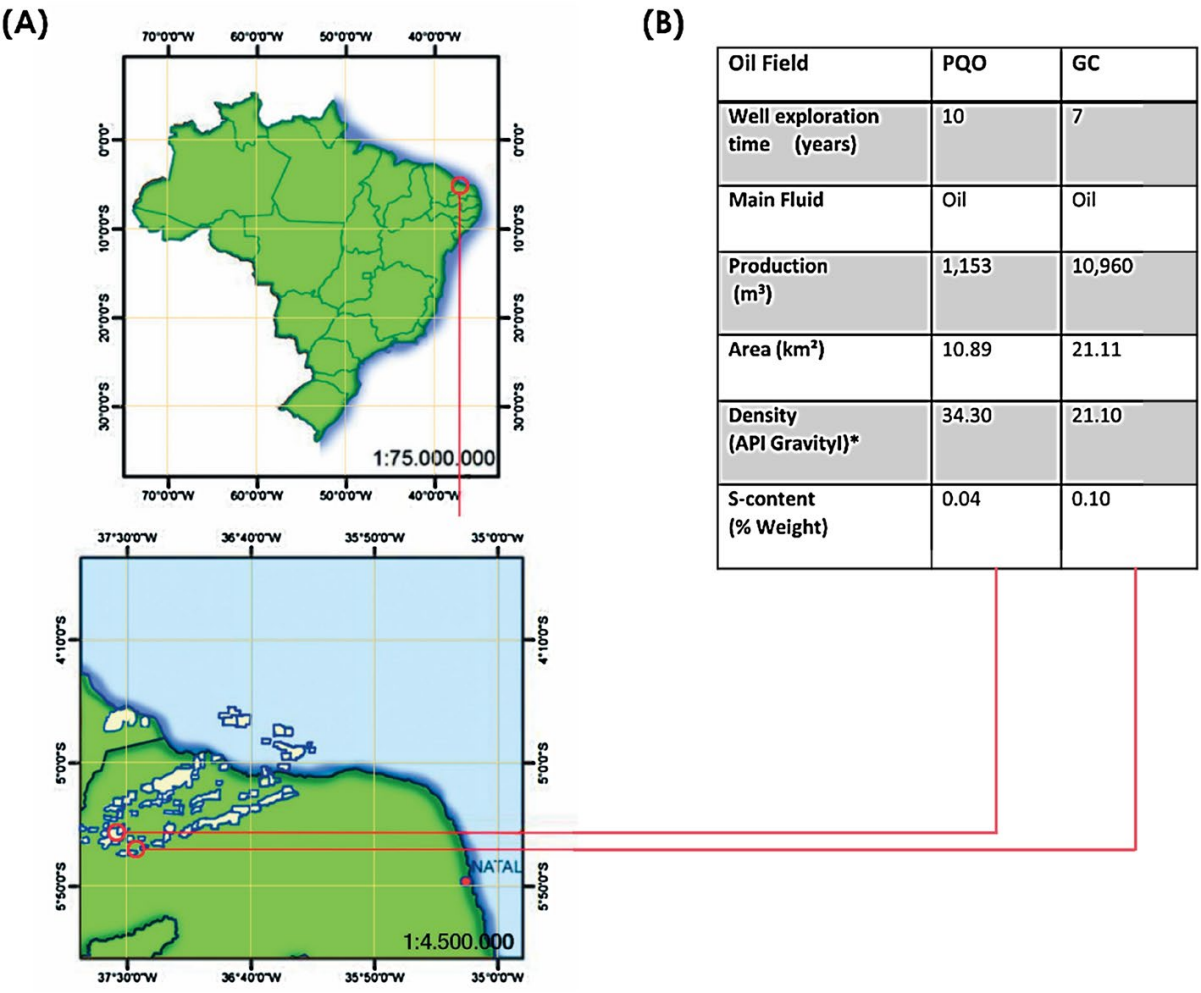
**(A)** Geographical location **(B)** Characteristics of Periquito (PQO) and Galo de Campina (GC) onshore oilfields from Brazil.

Produced water was sampled from the Galo de Campina (GC) and Periquito (PQO) oilfields. The Galo de Campina oilfield is located in the Potiguar Basin (Lat -05:26:44,532 Long -37:36:16,235), Rio Grande do Norte State, about 50 km from the urban centre of Mossoró city. The Periquito oilfield is also located in Rio Grande do Norte State, in the southwest part of the Potiguar Basin (Lat -05:30:01,409 Long -37:26:06,855). Samples (50 mL) of produced water were collected from the oil treatment separator unit of each oilfield using a top fed sterile airtight plastic container. Produced water samples were stored refrigerated between at 0–4 ºC under aseptic airtight conditions until enrichment. From each of the Galo de Campina and Periquieto oil fields, 3 technical replicas were collected for laboratory processing. Each replica was divided in three sub-samples for each medium. Major characteristics of the two oilfields are in shown in Fig. 1.

### SRB selective media

3 different growth media were added to the produced water samples to encourage the growth of SRB, the media were: (i) Postgate B (K_2_HPO_4,_ 1.0/NH_4_Cl, 2.0/CaSO_4_ 0.2H_2_O, 1.3/MgSO_4_ ·7H_2_O, 4.0/Lactic acid (88%) 2.7); Postgate C^35^, Sodium Lactate, 6.0/Na_2_SO_4,_ 4.5/NH_4_Cl, 1.0/Yeast Extract, 1.0/K_2_HPO_4,_ 0.5/C_6_ H_5_Na_3_O_7_ 0.2H_2_ O, 0.3/; CaCl_2_ 0.6H_2_O, 0.06/MgSO _4_ ·7H_2_O, 0.06/FeSO_4_ 0.7H_2_O,); 0.004 and Baars^36^, MgSO _4_ ·7H_2_O, 4.096/C_6_ H_5_Na_3_O_7_ 0.2H_2_ O, 5.7;/CaSO_4_, 1.0 NH_4_Cl, 1.0/K_2_HPO_4_, 0.5/Sodium lactate 4.5 ml/Yeast extract 1.0/Fe(NH_4_)_2_ (SO_4_)_2_, 6.72 g 50 ml 5 ml, for 1000 ml medium). These media are cited in studies on SRB. Using a sterile syringe, 10 ml of media plus 1 ml of produced water samples was injected into a vial and purged with nitrogen free oxygen gas for 2-min, before being clamped with a rubber and aluminium cap. The sealed tubes were stored for 20 days at 30° C under anaerobiosis, using anaerobic atmosphere generation bags and Gaspak jars (Merck Ltd).The anaerobic system contains a palladium catalyst and an indicator of anaerobiosis (methylene blue). The culture media contains the oxidation–reduction indicator resazurin, and an envelope containing sodium borohydride and sodium bicarbonate (Merck Ltd). For each oil field nine sub samples were investigated, 3 of each type of medium for each oilfield. After 20 days, bacteria from the 18 samples were collected by centrifugation for DNA extraction. Tubes under the same conditions containing just the medium without produced water provided the negative control.

### DNA extraction and molecular identification of bacteria

Genomic DNA was extracted with the Wizard Genomic DNA Purification Kit (PROMEGA) and quantified using QUBIT fluorometer (Thermo Fisher Scientific). DNA purity and quality were evaluated by 1% agarose gel electrophoresis containing SYBR Safe DNA gel stain (Life Technologies) at 90 V in 0.5X TBE buffer for 1 h and visualized by transillumination under ultraviolet light. 16S rDNA was PCR amplified.. The negative controls (media without produced water) did not show microbial growth and after DNA extraction no DNA was seen on gels and PCR amplifications were negative, these blank samples were not sequenced. The V3 and V4 variable region of the 16S rDNA was amplified by PCR, using the primers 338F^37^ and 806R^38^ with a barcode on the forward primer, in a 30 cycle PCR using the HotStarTaq Plus Master Mix Kit (Qiagen, USA) under the following conditions: 94 °C for 3 min, followed by 28 cycles of 94 °C for 30 s, 53 °C for 40 s and 72 °C for 1 min, after which a final elongation step at 72 °C for 5 min was performed. After amplification, PCR products were checked on a 2% agarose gel to determine the success of amplification and the relative intensity of bands. Samples were purified using calibrated Ampure XP beads. Purified PCR product was used to prepare the DNA library by following the Illumina TruSeq DNA library preparation protocol (Illumina, Sab Diego, CA, USA). Paired-end sequencing (2 × 300) was performed at MR DNA (www.mrdnalab.com, Shallowater, TX, USA) on a Illumina MiSeq (Illumina, Sab Diego, CA, USA) following the manufacturer’s guidelines. After sequencing, the two reads from the paired end sequencing were joined together after q25 trimming of the ends, with the MrDNA (www.mrdnalab.com, Shallowater, TX, USA) pipeline prior to further analyses.

### Sequence identification

The raw joined sequences were processed using Mothur v.1.36.1 software^39^. The sequences were trimmed using trim.seqs command, with the following parameter: qwindowaverage = 30, qwindowsize = 50, maxambig = 0, minlength = 410, maxlength = 500. The sequences were then aligned using the Silva database (v132) as a reference^40^ and the resultant alignments were submitted to screen.seqs and filter.seqs to remove sequences with bad alignments and to remove uninformative columns of the alignment. The sequences where then pre-clustered using the command pre.cluster with parameter diffs = 3. Chimeras were detected with the chimera.uchime command, which uses the Uchime software^41^, chimeric sequences were removed from further analyses. The remaining sequences were then classified using classify.seqs command, with RDP database as reference and a bootstrap cut-off of 80. Sequences classified as chloroplasts, mitochondria, and those not assigned to any kingdom were removed, No eukaria or archaea sequences were seen in the samples. The resultant sequences, were used as input for the construction of a distance matrix and for clustering the sequences into operational taxonomic units (OTUs), with a cut-off of 3% of dissimilarity. The samples were then randomly normalized to the same number of sequences (n = 46,736) for biodiversity analyses. Then the taxonomy summary was used to identify the bacterial compositions of each sample, and the OTU distributions were used to calculate the diversity indices (Chao and Shannon index).

### Statistical analysis

Regarding the nature of data distribution, all indices showed a normal distribution. The final files generated by Mothur were imported into R (v4.0)^42^ where the microbiome data was processed through the Phyloseq^43^ and Microbiome R^44^ packages. The Non-Metric Dimensional Scaling (NMDS) analysis was used to visualize the (dis)similarities between the samples, with NMDS ordinations in two dimensions calculated from a Bray–Curtis dissimilarity matrix. The quality of the NMDS representation was evaluated through the generated stress value; considering values < 0.2 as good representation of the data^45^. To verify the separation of the groups observed in the NMDS, a Permutational Multivariate Analysis of Variance (PERMANOVA) was performed. Finally, to ensure that the result of PERMANOVA was not affected by dispersions in the groups, an analysis of multivariate homogeneity of group dispersion (MHGD) was carried out. Differential OTU abundance between oil fields (Galo de Campina and Periquito) was analyzed using the DESeq2^46^. The outputs were adjusted using the Benjamini-Hochberg (BH) method and the values of differential abundances were measured in log2-Fold-Change. A taxon was considered significant when it met the criteria of adjusted p-value < 0.05 and absolute value of log2-Fold-Change > 2.0. Finally, the selected taxa were assessed separately using the t-test with adjustment of the p-value using the BH method^47^. All statistical analyses were performed using R (v4.0)^42^ with the support of the vegan package^48^. The BCM, PERMANOVA and MHGD analyses were performed using the functions vegdist(), adonis() and betadisper() from the vegan package.

## Results and discussion

### Bacterial biodiversity of produced water

The sequence data were deposited in the NCBI Sequence Read Archive (SRA) and are available under accession number SRP149784. In this study, 4.206.240 sequencing reads were generated, all the sequences studied were bacterial.. From 4,206,240 reads a total of 841,248 sequences were analyzed the remainder were trimmed. The bacterial diversity indices for produced water enriched for SRB for the Galo de Campina (GC) and Periquito (PQO) oil fields are shown in Table 1.

**Table 1:** Biodiversity of the Produced Water samples.

Sample	Oil Field	OTUs	Chao	Shannon
GCa-BA	Galo de Campina	101	176	0.98
GCa-PB	Galo de Campina	110	270	0.74
GCa-PC	Galo de Campina	119	275	0.96
GCb-BA	Galo de Campina	108	244	0.86
GCb-PB	Galo de Campina	95	169	0.72
GCb-PC	Galo de Campina	126	280	0.86
GCc-BA	Galo de Campina	143	405	1.29
GCc-PB	Galo de Campina	130	380	1.08
GCc-PC	Galo de Campina	150	370	1.42
PQOa-BA	Periquito	119	253	1.06
PQOa-PB	Periquito	119	272	1.17
PQOa-PC	Periquito	136	484	1.12
PQOb-BA	Periquito	144	443	0.99
PQOb-PB	Periquito	133	276	1.17
PQOb-PC	Periquito	128	349	0.91
PQOc-BA	Periquito	120	243	0.91
PQOc-PB	Periquito	101	231	0.97
PQOc-PC	Periquito	93	179	0.86

BA = Baars medium, PB = Postgate B and PC = Postagate C; Galo de Campina oilfield (GC) and Periquito oilfield (PQO) a,b, and c sub-sample number.

Based on a 3% dissimilarity cut off, a total of 992 OTU´s were defined and compared. The number of OTUs (species richness/alpha diversity) varied between 95 and 150 at Campo de Galo and between 93 to 144 at Periquito. Chao and Shannon values were evaluated by Shapiro–Wilk for normality and homogeneity, having satisfied those requirements, the Student's t test was used for analysis of variance. Comparisons of the Chao (*P* = 0.69) and Shannon (*P* = 0.75) indices were made and there were no significant differences in biodiversity between the oil fields. At the 3% sequence dissimilarity cut off, there was no significant difference between the different culture media analyzed.

**Figure 2:**
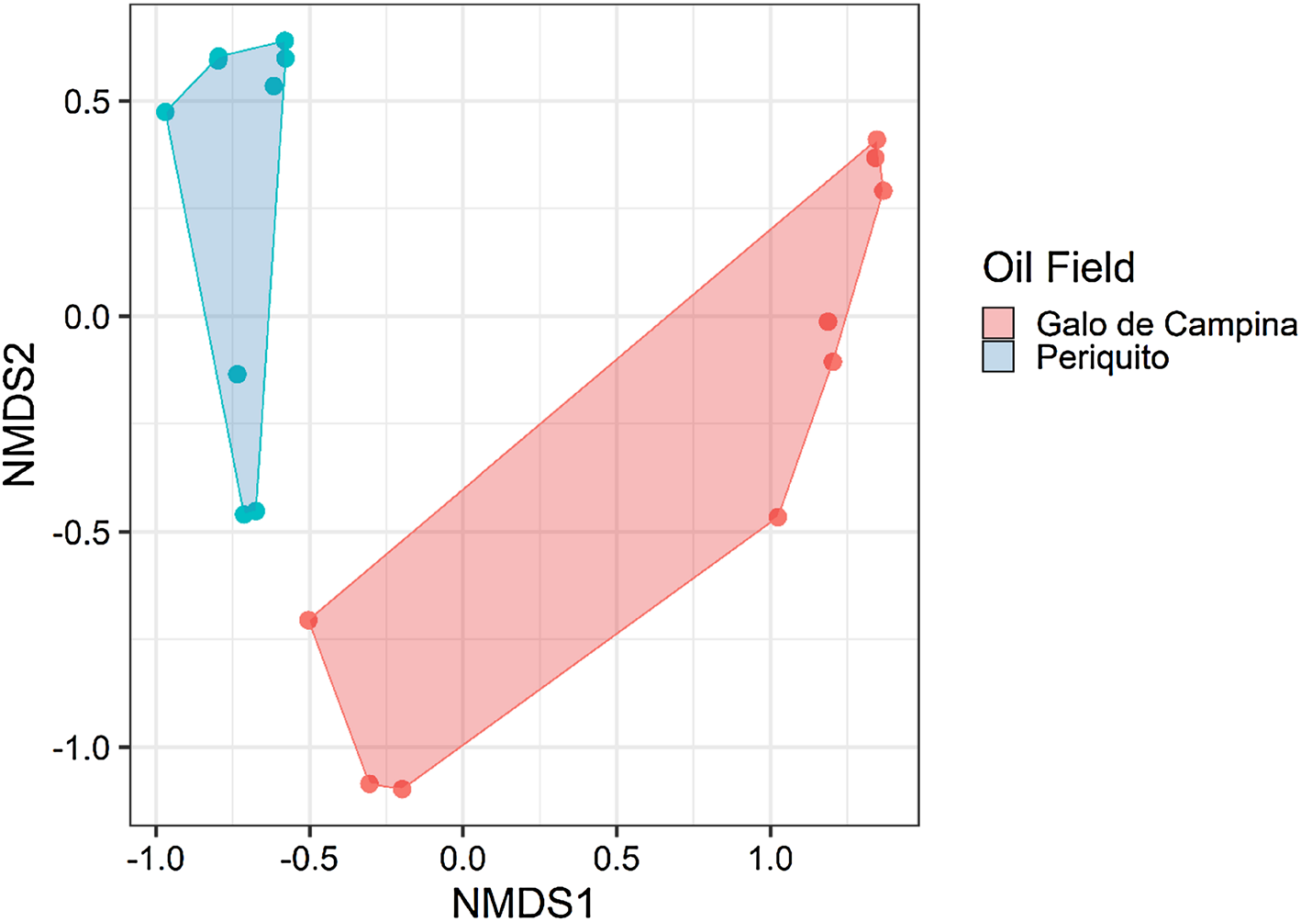
Non-metric multidimensional scaling analysis (nMDS) of replicas from the Galo de Campina (red) and Periquieto (blue) oilfields (n = 9).

NMDS (Fig. 2) and Bray–Curtis produced a stress = 0.06. It was possible to observe a good clustering and a separation by oil field. The GC samples clustered in the positive NMDS1 portion, while GC samples were found in the negative NMDS1. NMDS results were further supported by a PERMANOVA analysis (*P* = 9.99 e-05, R^2^ = 0.94). demonstrating that there is a significant difference between the groups analyzed (Galo de Campina and Periquito oil fields). This result was supported by the homogeneity of variances (*P* = 0.09) which revealed that there is no inter-individual variation. Overall, these results suggest that there is a real difference in SRB between the oil fields. The results of the BCM analysis indicate that differences seen between samples is explained by differences between the two oilfields.

### Taxonomic distributions

No differences in bacterial diversity were observed as a result of using three different enrichment media. An anlysis of media composition reveals that they are similar. The dominant phyla, orders, classes were dominant regardless of which of the three medias was used. The three SRB enrichment media were used to recover a diverse collection of bacteria with the desired metabolic trait, with hindsight, one would have sufficed^49^.

Starting at the phylum level, the distribution of phyla seen in the produced water enriched to detect SRB at both the Galo de Campina and Periquito oilfields was dominated by the Proteobacteria, 93.8%, and Firmicutes with 6.2%. While this study purposefully enriched and selected for SRB it is interesting to report that in other studies of total bacterial diversity from produced water and not SRB enriched, show that phylum level results are similar and global. Belgini et al.^50^ identified Proteobacteria *(Brevundimonas* sp, *Sphingobium* sp., *Sphingopyxis* sp., *Sphingomonas xenophaga*, *Achromobacter* sp., *Delftia tsuruhatensis*, *Aeromonas* sp., *Pseudomonas* sp., *Rheinheimera* sp. and *Serratia* sp.), Firmicutes (*Bacillus* sp. and *Lysinibacillus* sp.) and Bacteroidetes (*Chryseobacterium* sp., *Flavobacterium* sp.) as the dominant phyla in samples from the Gabriel Passos Refinery (REGAP) in the city of Betim (MG, Brazil). In a study of produced water by de Sousa Pires^51^, from an offshore oil field in southeast Brazil, the bacterial community was dominated by sequences associated with SRB and within the Proteobacteria (specifically Desulfobacterales), Firmicutes and Bacteroidetes. A similar pattern was found in produced water from the Diyarbakır oil Fields in Turkey^52^. In Turkey, the Proteobacteria were the predominant phylum in all samples. In a study from the Mae Soon Luang Field, Fang Basin, Thailand^53^, Proteobacteria, Firmicutes and Actinobacteria dominated the bacterial communities in most samples. Similar reports are seen from Texas, USA by Santillan et al.^13^, Li et al.^54^ and from China by Lan et al.^55^. Silva et al.^31^ studied the microbial communities in petroleum samples from Brazilian offshore oil fields and found that the most abundant phylum was the Proteobacteria, followed by the Firmicutes (Bacilli and Clostridia). Despite the differences in geographical locations of oilfields and methods used to investigate them, it is interesting to note that these two phyla always dominate. This is perhaps not surprising if we consider that conditions within an oil reservoir will strongly select for specific groups of bacteria that can survive and evolve in such environments.

**Figure 3:**
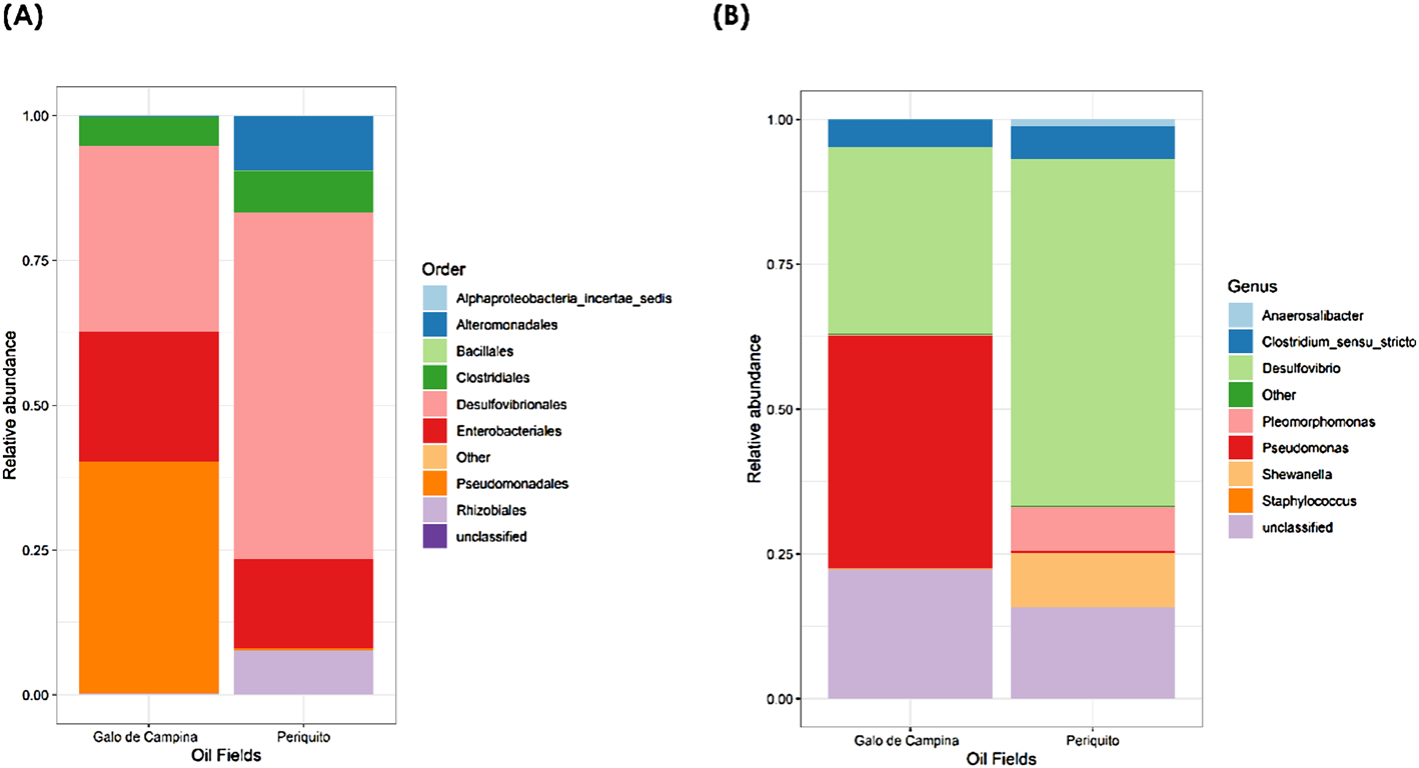
(**A**) The relative abundance of bacterial orders found in produced water enriched for SRB from the Galo de Campina and Periquito oilfields. (**B**) The relative abundance of bacterial genera found in SRB enriched produced water from the Galo de Campina and Periquito oil fields.

The main orders detected in the produced water from Galo de Campina and Periquito were Desulfovibrionales, Pseudomonadales, Enterobacteriales, Clostridiales, Alteromonadales, Rhizobiales and Bacillalles and their distributions are shown in (Fig. 3A). Note at the order level it was possible to identify and classify all sequences. Li et al.^54^, in their work on biofilms in water injection systems in the Daqing oil field (China), also identified Pseudomonadales, Enterobacteriales, Clostridiales and Desulfovibrionales and associated them with hydrogen sulphide production, biofilm formation and biocorrosion^12,13,27^. Desulfovibrionales strains were present in all samples and dominant in both oil fields accounting for 46% of all sequences. The majority of bacteria in this order are SRB and are responsible for biocorrosion. Their presence in these samples might explain problems with hydrogen sulphide at the Periquito oilfield. Pseudomonadales was the dominant order in samples from the Galo de Campina oilfield and Enterobacteiriales strains were present in both oil fields. The Enterobacteriales and Alteromonadales reported here have been previously associated with oil environments^56,57^.

Genus level diversity of the SRB is shown in Fig. 3B and the main genera are summarized in Table 2. *Desulfovibrio* is the main and dominant genus in produced water from both the Galo de Campina (16,08%) and Periquito (29,7%) oilfields. Next *Pseudomonas* in Galo de Campina (19,88%) and *Shewanella* in Periquito (4,69%). Some bacterial sequences were too divergent for classification at the genus level but their order level identity is shown in Fig. 3A. It is possible that the 300 bp sequence length explains this lack of taxonomic resolutionç but also one can infer, but not confirm, that novel SRB genera are present in both oilfields. *Clostridium* were detected in low numbers from both oil fields. The Postgate C media, favoured the detection of *Desulfovibrio* over the other media though this trend was not statistically significant at P 0.95. Here we used three media with lactic acid as a carbon source but there are other SRB metabolic types with different types of electron donorsç^18^ as such, our study might under represent the SRB diversity. A wide range of organic acids including acetate, propionate, butyrate, pentanoate, and hexanoate have been found in oil reservoirs^58^ and are alternative electron donors. Bacteria of the genera *Desulfovibrio* and *Clostridium* are well described producers of hydrogen sulphide and involved in the corrosion of metallic structures^28,59^. According Christman et al.^60^ Clostridiales’ sequences are often reported in produced water, however, their role is uncertain because they might not be actively producing H_2_S and simply present in a dormant sporulated form.

**Table 2:** Most abundant genera found in Galo de Campina e Periquito oilfield.

Genera	Galo de Campina (%)	Periquito (%)
*Desulfovibrio*	16.1	29.9
*Pseudomonas*	19.9	0.18
*Clostridium*_sensu_stricto	2.40	2.81
Shewanella	0.07	4.69
Pleomorphomonas	0.07	3.77

*Unclassified genera from Galo de Campina is 11,1% and Periquito 7,62.

*Pseudomonas* sequences were detected in much larger numbers at the Galo de Campina oilfield in comparison with Periquito and this could be a possible biomarker for that site. *Pleomorphomona* were all but exclusively identified at Periquito and could serve as a biomarker there. The genus *Pleomorphomona* is not normally associated with produced water. On the other hand the genera *Desulfovibrio, Pseudomonas* and *Clostridium* are routinely found in produced water samples^61^. Strains from the genus Desulfovibrio are established sulphide producers and thus represent a major concern for the oil and gas industry^62,63^. What stands out immediately from the results is the importance of the *Desulfovibrio*^64,65^. *Desulfovibrio* are known to cause corrosion, hydrogen sulphide production, souring and biofouling within the operational plants^71,72^. More than 220 species from 60 genera of SRB have been described; of which, the most commonly isolated mesophilic SRB from produced water are from the *Desulfovibrio* genus^64^. Also of note at the genus level were the *Pseudomonads* at Galo de Campina, and the large number of (unidentified at genus level) Enterobacteriales. The frequent detection of *Pseudomonas* and unclassified Enterobacterales in the enrichments from the Galo de Campina are also of high research interest. To further characterize these taxa it will be necessary to isolate and properly describe these produced water taxa. The genera *Pleomorphomonas* and *Shewanella* were more abundant in samples from the Periquito oil field. *Pleomorphomonas* was first described in 2005 by Xie and Yokota^66^ and is genus composed of gram-negative, pleomorphic, nitrogen-fixing, non-spore-forming, non-motile rods and first isolated from rice fields^67,68^. At the time of revision, there is no data correlating the presence of strains of this genus with problems in oil industry. *Shewanella* are facultative anaerobic, gram-negative, motile and rod-shaped bacteria, most of which have been isolated from marine environments, such as seawater, marine sediments or sand, tidal flats or marine invertebrates. Some species have, however, been isolated from clinical samples, oilfield fluids, activated sludge and coal-mine sludge^69,70^. *Shewanella* strains were described as a potential hazard to the oil industry causing souring of crude oil^71^.

**Figure 4:**
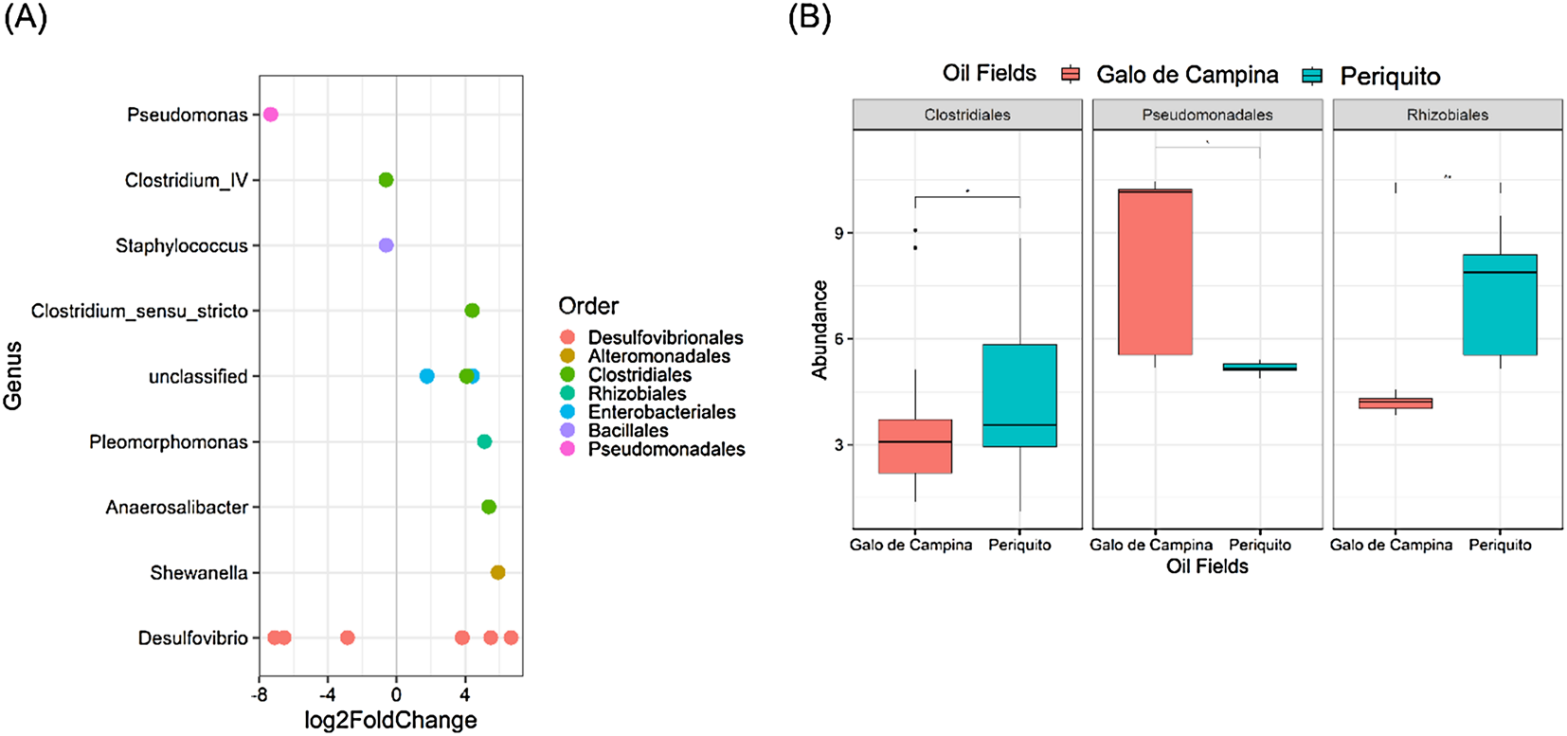
Differential abundance of OTUs between oil fields using DESeq2. (**A**) Bar plot showing the log2foldchange of abundance at the Order and Genus level. (**B**) OTUs that presented abundances with significant variations (adjusted p-value < 0.05) with respect to oil fields.

A deeper analysis at the genus level was undertaken to evaluate if there were significant differences in the distribution of genera between the oilfields, results are shown in Fig. 4.

In Fig. 4A, the DESeq2 bar plot shows on the x-axis the log2-fold-change of abundance of the orders that showed the greatest variation between oil fields. The DESeq2 method selected the 8 most relevant genera to differentiate the two oil fields. After applying the adjusted p-value < 0.05 and log2FoldChange > 2 criteria, 3 orders were selected, namely Clostridiales, Pseudomonadales and Rhizobiales. Figure 4B illustrates the variation of these OTUs in each oil field, where it can be seen that there was a significant variation (BH adjusted p-value < 0.05) between the two fields analyzed. It is interesting to note that Clostridiales and Rhizobiales showed greater abundance in the Periquito field, while Pseudomonadales was more abundant in the Galo de Campina field.

The limited differences between the oil fields is in agreement with the enrichment methodology; and, work carried out by Kim et al.^72^ who analyzed the microbial community in produced water collected from four geographically distant oil reservoirs and reported highly similar microbial communities in oil reservoirs in geographically distant locations. One can infer that geographical location is a less important driver of diversity in produced water than are oil reservoir conditions (temperature, pressure, pH) and that similar conditions in oil reservoirs naturally select similar groups of organisms. The order Rhizobiales is constantly found in microbial communities related to oil^73–77^ and according to Angelova et al.^73^, strains from this genus are involved in polymer degradation, biofilm formation, and sulphur oxidation.

The data obtained here from SRB enriched produced water and with large scale NGS sequencing is consistent with the results reported in literature based on isolation methods and on nucleic acid methods where organisms were neither enriched nor isolated^29,31,78^. Other groups of microorganisms such as the extremophile halophiles and halotolerant microorganisms could be present in produced water after hydraulic fracturing. Although these microorganisms were not the focus of our study, it is interesting to note their presence in hypersaline environments. *Halanaerobium* spp, *Halanaerobium*, *Marinobacter, Methanohalophilus, Methanolobus* and members from Halobacteroidaceae and Halomonadaceae would not be unexpected in an offshore hydrocarbon environment^76,79–82^ . It is only at the genus level that differences between the oil fields become evident. Bacterial strains of the genus *Pseudomonas* are producers of extracellular polymeric substances that form biofilms within oil facilities^25^. The presence of mesophilic *Pseudomonas* strains that are sensitive to high temperatures is not believed to originate from pristine oil reservoirs. Their presence in systems with high temperature oil wells is likely to follow flooding with cooler produced water, and contamination with *Pseudomonas* strains that are very versatile heterotrophs with the competitive capability to survive and form biofilms in pipes with oil/water mixtures^29^. Zdanowski et al.^83^ analyzed the anaerobic microbiome of subglacial samples and compared the phylogenetic diversity of native samples with enriched ones. Enrichment was done by incubating native sediments in Postgate C medium for 8 weeks using airtight bottles to emulate subglacial conditions. They found that *Psychrosinus, Clostridium, Paludibacter, Acetobacterium, Pseudomonas, Carnobacterium,* and *Desulfosporosinus* were more abundant in the enriched medium: *Pseudomonas* and *Carnobacterium w*ere found only in the enriched medium. Thus, the authors suggest that it may be that bacteria of the genus *Pseudomonas* have proliferated under enrichment conditions, depleting available oxygen in the early stages of the process, and then helping anaerobes to develop. In a study by Guo et al.^84^, the authors demonstrated that *Pseudomonas* strain sp. C27 has an enzymatic system to perform sulfide removal. The authors cultivated the C27 strain in an anaerobic environment and demonstrated that the sulfide metabolism occurred through the expression of succinate dehydrogenase, iron–sulfur protein oxidoreductase, serine hydroxymethyl transferase, and iron superoxide dismutase. Brahmacharimayum and Ghosh^85^ analyzed the removal of sulphate in an anaerobic environment and reported that a *P. aeruginosa* strain was dominant in the consortium and that it was involved in reducing Sulphate. Tüccar et al.^52^, found *Pseudomonas* as the dominant genus in produced water from Diyarbakir oil fields in Turkey.The authors suggested that these strains may have been inoculated into the oil reservoirs through the injection of produced water and that strains adapt to the conditions of the reservoir to survive. Several studies have found *Pseudomonas* in oil reservoirs^86–92^, and according to Cui et al.^93^, *Pseudomonas* and *Acinetobacter* are genera that can effectively use crude oil as a carbon source, being able to survive and reproduce at the oil–water interface. Species of the genus *Pseudomonas* are facultative anaerobes capable of performing nitrification and nitrate reduction using various carbon substrates^94,95^. In a study by Braun and Gibson^96^, the authors reported on two bacterial strains of the genus *Pseudomonas* that were able to degrade, under anaerobic conditions, 2-aminobenzoate (anthranilic acid) to CO2 and NH_4_ +.  According to Cai et al.^97^, in the petroleum area, despite the fact that the oil is considered a hostile and toxic environment for these microorganisms, there is now a lot of evidence that demonstrates the presence and activity of these microbes in the crude oil. Cai et al.^97^ analyzed the microorganisms found in oil and water samples and showed that the genus *Pseudomonas* dominated the oil samples^98^. The *Pseudomonas* may well soon be considered at problematic as the *Desulfovibrio* ^99^*.*

## Conclusion

DNA sequencing analysis of over 800 000sequences from the Galo de Campina and Periquito oil fields detected a dominant phylum, the Proteobacteria (93.8%) which was followed by the Firmicutes (6.2%). These phyla are typically described in all global studies of reservoir and produced water bacterial biodiversity. The rDNA data in this study therefore supports previous findings based on isolation and direct DNA extraction studies. Seven major orders were detected, Clostridiales and Bacilliales belonging to the phylum Firmicutes, and Desulfovibrionales, Pseudomonadales, Enterobacteriales, Alteromonadales and Rhizobiales that belong to the phylum Proteobacteria. Among the genera detected, we noted that *Pseudomonas*, *Desulfovibrio* and *Clostridium* were the dominant genera, and that bacteria from these genera are routinely isolated from samples of produced water. They are known to be involved in biofilm formation processes, production of hydrogen sulphide and corrosion of metallic structures. For these two onshore oil fields we now know which bacteria in the produced water responded to the experimental conditions and that could possibly respond to attempts to manage those bacteria. To address the challenge of SRB at these oilfields and elsewhere requires monitoring SRB communities and biocides that target specific SRB groups. Microbial communities are both the problem and a solution to corrosion and souring; to decreasing maintenance and operating costs as well as to minimising negative environmental impacts in Brazil and elsewhere. The next step is to target specific problematic strains and employ targeted biocides that are more efficient at eradicating them and are less toxic to the wider environment than chemical alternatives.
